# Ground Mobile Robots for High-Throughput Plant Phenotyping: A Review from the Closed-Loop Perspective of Perception, Decision, and Action

**DOI:** 10.3390/plants15081218

**Published:** 2026-04-16

**Authors:** Heng-Wei Zhang, Yi-Ming Qin, An-Qi Wu, Xi Xi, Pingfan Hu, Rui-Feng Wang

**Affiliations:** 1 College of Engineering, China Agricultural University, Qinghua East Road No. 8, Haidian, Beijing 100083, China; hwzhang@cau.edu.cn; 2 International College Beijing, China Agricultural University, Qinghua East Road No. 8, Haidian, Beijing 100083, China; ming2919@cau.edu.cn; 3 Yantai Institute, China Agricultural University, Qinghua East Road No. 8, Haidian, Beijing 100083, China; ongkingu@cau.edu.cn; 4 College of Economics and Management, China Agricultural University, Qinghua East Road No. 8, Haidian, Beijing 100083, China; xixi@cau.edu.cn; 5 Artie McFerrin Department of Chemical Engineering, Texas A&M University, College Station, TX 77843, USA; pingfanhu@tamu.edu; 6 Department of Crop and Soil Sciences, College of Agriculture and Environmental Sciences, University of Georgia, Tifton, GA 31793, USA

**Keywords:** high-throughput plant phenotyping, unmanned ground vehicles, mobile robotics, multimodal sensing, field phenotyping, precision agriculture, deep learning

## Abstract

High-throughput plant phenotyping (HTPP) is increasingly limited by the mismatch between the need for field-relevant, fine-grained phenotypic information and the restricted capability of conventional observation platforms under complex agricultural conditions. Ground mobile robots are emerging as the key carrier for resolving this gap because they combine close-range sensing, autonomous mobility, and physical interaction within real field environments. In this paper, a structured scoping review is presented using a closed-loop perception–decision–action pipeline as the organizing principle. Within this framework, recent advances are synthesized from the perspectives of multimodal fusion, localization-aware sensing, motion planning, deep-learning-based phenotypic analysis, active observation, robotic intervention, and edge deployment. The review further clarifies the complementary roles of Unmanned Aerial Vehicles (UAVs), Unmanned Ground Vehicles (UGVs), and air–ground collaboration in multiscale phenotyping workflows. Beyond summarizing technologies, the article provides three concrete deliverables: a structured taxonomy of mobile phenotyping systems; comparative tables covering sensing modalities, localization/navigation methods, and AI models; and a research agenda linking technical progress to field deployability. The synthesis highlights four persistent bottlenecks, namely environmental generalization, annotation scarcity, limited standardization and reproducibility, and the gap between advanced models and agricultural edge hardware. Overall, ground robots are identified not merely as sensing platforms, but as the central system architecture for advancing mobile phenotyping toward autonomous, fine-grained, and field-deployable operation.

## 1. Introduction

Global agricultural production is under growing pressure from two converging forces: steadily rising food demand and escalating climate risk [1,2]. United Nations projections indicate that the global population will reach approximately 9.7 to 10 billion around 2050, sustaining long-term growth in demand for food and feed [1]. At the same time, the Sixth Assessment Report of the Intergovernmental Panel on Climate Change warns that climate warming and the increasing frequency of extreme weather events have already become tangible threats to global food security and will further intensify the vulnerability of agricultural systems in the future [2,3]. Against this backdrop, improving resource-use efficiency and system resilience through crop breeding has become a key technological pathway for supporting sustainable agricultural development [4,5,6,7].

Meanwhile, the rapid development of artificial intelligence (AI), machine learning (ML), and deep learning (DL), together with their successful applications across multiple domains [8,9,10,11,12,13,14,15,16], has created new opportunities for automated, scalable, and fine-grained phenotypic analysis in agriculture [17,18,19,20]. In this context, High-Throughput Plant Phenotyping (HTPP) has emerged as a key technological framework for linking genomic information with field-relevant phenotypic evidence. Its central aim is to quantitatively characterize plant morphology and physiological processes across large populations, diverse environments, and multiple time points, thereby supporting both breeding selection and precision field management [1,21,22].

Although existing HTPP technologies have, to some extent, alleviated the low efficiency and inconsistency associated with traditional manual measurements, their applicability in complex field environments remains limited. Automated greenhouse systems and conveyor-based platforms in controlled environments can provide highly consistent imaging conditions and standardized data acquisition, but they cannot faithfully reproduce the fluctuating illumination, wind disturbance, terrain variation, and population heterogeneity of open-field settings. Consequently, the extent to which their phenotypic outputs can be extrapolated to real agricultural environments remains constrained [4]. In contrast, UAVs, with their rapid response, broad coverage, flexible deployment, and relatively low cost [23,24], have become important platforms for temporal remote sensing in breeding trials [25,26]. Equipped with RGB, multispectral, and thermal infrared sensors, UAVs can efficiently capture canopy-scale traits related to crop growth, stress responses, and certain yield-associated indicators [27,28,29]. However, as high-throughput phenotyping shifts from simply acquiring data to acquiring data that are sufficiently detailed and directly actionable for decision-making, the central challenge is no longer data collection per se, but rather the mismatch among observation precision, system robustness, and end-to-end automation under complex field conditions [3,30].

More specifically, current mobile phenotyping systems still face several major constraints. Firstly, because UAVs primarily rely on top-view or oblique-view observations of the canopy, they are highly susceptible to occlusion in dense plantings or tall crops, which limits their ability to stably measure lower-canopy and organ-level traits. As a result, traits such as stem architecture, ear or fruit morphology, lower-leaf posture, and early concealed symptoms of pests and diseases are difficult to capture reliably [25,28,31]. In contrast, although Unmanned Ground Vehicles can acquire high-resolution, close-range, and multi-view data, they generally lag behind UAVs in operating speed, area coverage efficiency, and adaptability to complex terrain, making it difficult for them to independently satisfy the throughput and timeliness requirements of large-scale breeding trials [32]. Secondly, traditional cart-based platforms and manual close-range sampling can enter crop rows and collect data at high spatial resolution, but they remain labor-intensive, low-throughput, and highly dependent on operator experience and workflow consistency, limiting their suitability for large-scale standardized field phenotyping [1,30]. Thirdly, many current phenotypic analysis methods and system pipelines are still validated primarily on offline datasets, and their engineering readiness for real field deployment remains insufficient. Under challenging conditions such as abrupt illumination changes, wind-induced leaf motion, muddy terrain, weed interference, and long-term unattended operation, substantial weaknesses persist in perception robustness, closed-loop data transmission and processing, online decision-making, and long-term operational stability. These limitations have become major barriers to the integrated deployment of algorithms, platforms, and practical applications [30,33,34,35].

Representative studies synthesized in this review have reported measurable gains from multimodal or multi-view integration under specific task settings—for instance, multimodal fusion networks have achieved mAP50 improvements of 2.3–6.9% in occluded-target detection [36,37], and UAV–UGV collaborative sensing has yielded up to 13.6% higher accuracy in phenological-stage recognition compared with single-view baselines [38]—however, maintaining such performance under canopy occlusion, illumination variability, and complex field conditions remains a major engineering challenge.

Against this background, and in pursuit of precise phenotypic analysis that is multi-scale, comprehensive, and deployable in practice, research is gradually moving away from single-platform observation toward a mobile phenotyping paradigm centered on ground robots and complemented by air–ground collaboration [34,39]. Compared with UAVs, UGVs can operate within crop rows and beneath the canopy while carrying heavier and more complex sensor suites, such as multi-view imaging systems, LiDAR, and close-range spectrometers. They are therefore better suited for organ-level structural trait measurement, fine-grained observation under occlusion, and three-dimensional reconstruction [40,41]. Existing studies have already demonstrated the significant potential of autonomous field robots for high-throughput, high-resolution 3D phenotyping, while also revealing the navigation, perception, and system integration constraints associated with deployment in real agricultural environments. Together, these findings suggest that combining close-range 3D sensing with autonomous mobility is a promising direction for overcoming occlusion and enabling the quantification of complex structural traits [33,42]. At the same time, air–ground collaboration (UAV–UGV) offers a scalable workflow that balances coverage efficiency with information density: UAVs perform large-area scanning, anomaly detection, and task guidance, while UGVs conduct targeted close-range measurements and fine-grained information completion, thereby achieving a better balance between overall throughput and local precision [39,43,44].

Several reviews have addressed complementary aspects of this evolving landscape, yet collectively they leave three specific gaps unresolved. Reviews focused on UAV-based phenotyping [26,45] have substantially advanced the understanding of canopy-scale sensing but, by their aerial-platform orientation, do not address the near-canopy autonomous navigation, organ-level observation, and physical intervention capabilities that define the functional advantages of ground robots. The survey by Xu and Li [30], the most directly relevant ground-robot review, systematically documents platform configurations and sensor integration strategies; however, it predates the widespread emergence of foundation models such as the Segment Anything Model (SAM), generative artificial intelligence for data augmentation, and embodied intelligence frameworks, and it does not explicitly frame UGVs as closed-loop engineering systems integrating perception, decision-making, and action. Reviews of UAV–UGV collaboration [39,44] demonstrate the system-level value of air–ground integration but treat both platforms primarily as mobile sensor carriers, without systematically addressing the decision-making architectures, active observation strategies, robotic manipulation capabilities, or edge deployment constraints required for sustained autonomous field operation. Finally, the broader robotic phenotyping survey by Atefi et al. [22] provides a valuable cross-platform overview but does not distinguish the full closed-loop operational role of ground robots from other platforms, nor synthesize the convergence of deep learning, foundation models, and embodied intelligence that has since reshaped this domain. Taken together, three specific gaps remain: first, a robotics-centered framing of HTPP as a closed-loop perception–decision–action engineering problem rather than a collection of isolated sensing tasks; second, a systematic treatment of recent methodological advances, including foundation models, generative AI, 4D spatiotemporal reconstruction, and VLA-based embodied intelligence, within the agricultural phenotyping context; and third, a concurrent analysis of edge deployment engineering—covering computation, thermal management, communication, and energy—as an integral dimension of system design rather than an afterthought.

Building on this gap analysis, this review goes beyond a simple catalog of mobile platforms and sensor configurations and instead reframes high-throughput plant phenotyping, from a robotic systems perspective, as a closed-loop engineering problem of perception, decision-making, and action in complex field environments. To this end, the functional roles and complementarities of UAVs, ground robots, and their collaborative modes in multi-scale phenotypic data acquisition are first compared. The analysis then focuses primarily on ground mobile robots and conducts a structured scoping review of recent advances in the core technical framework spanning perception, decision-making, and action, with particular emphasis on multimodal sensing and autonomous localization, phenotype analysis driven by deep learning and generative artificial intelligence, and key enabling capabilities such as chassis control and active robotic manipulation. Edge computing, model lightweighting, and the practical challenges of field deployment under complex environmental conditions are further discussed. Finally, major bottlenecks, including environmental generalization, the scarcity of high-quality annotated data, and the lack of standardized benchmarks, are examined, and future directions in multi-robot collaboration, standard system development, and embodied intelligence for agriculture are outlined.

To make the scope and contribution of this review explicit, the main contributions are summarized as follows:

- A robotics-centered taxonomy is provided to organize mobile phenotyping systems within a closed-loop perception–decision–action framework.
- A comparative analysis is presented for UAVs, UGVs, and UAV–UGV collaboration, clarifying their complementary roles in multiscale phenotypic data acquisition.
- Recent advances in multimodal sensing, localization-aware perception, phenotypic AI, active observation/manipulation, and edge deployment are synthesized from the perspective of engineering robustness and field deployability.
- A future research agenda is outlined, with emphasis on environmental generalization, annotation scarcity, standardization and reproducibility, multi-robot collaboration, and embodied intelligence for agriculture.

### Review Methodology

To ensure transparency and reproducibility, this review was conducted following a structured scoping procedure informed by the Preferred Reporting Items for Systematic Reviews and Meta-Analyses (PRISMA 2020) guidelines, adapted to the engineering-oriented nature of ground mobile robotic phenotyping research. A comprehensive literature search was performed across Web of Science, Scopus, and IEEE Xplore, supplemented by Google Scholar for recent preprints. The temporal scope was primarily set from 2018 to 2026, capturing the peak period of deep learning and embodied intelligence integration in agriculture, while seminal earlier works were retained for foundational context. The search combined primary keywords, including (“unmanned ground vehicle” OR “UGV” OR “ground robot” OR “agricultural robot”) AND (“plant phenotyping” OR “high-throughput phenotyping” OR “crop phenotyping”), with secondary terms such as “multimodal sensing,” “SLAM,” “path planning,” “deep learning,” “foundation model,” “active observation,” and “edge deployment” used to refine the results. Boolean operators (AND/OR) were applied for flexible combination. Google Scholar-identified preprints were retained in the final synthesis only when they represented the most current or methodologically unique contributions not yet available in peer-reviewed form. Specific examples include very recent arXiv submissions on foundation models or Vision-Language-Action systems in agricultural robotics. Such cases were explicitly flagged during the qualitative quality appraisal described below.

**Figure 1:**
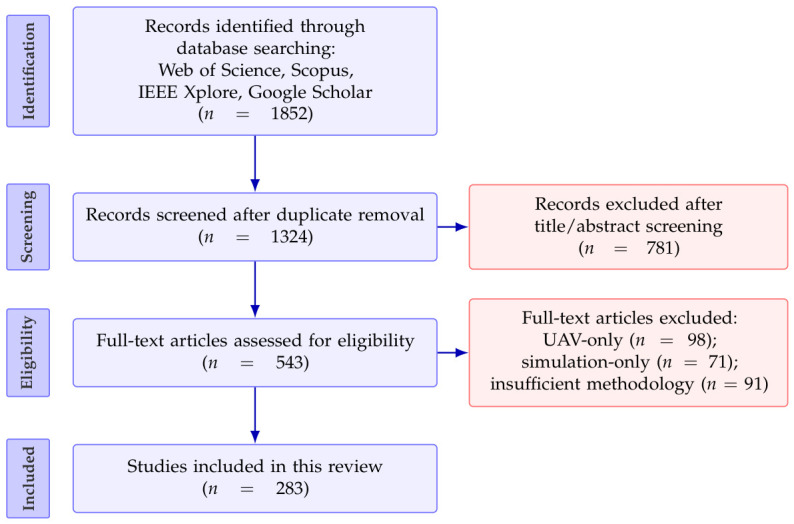
PRISMA-style flow diagram of the literature selection process for this scoping review on ground mobile robots for high-throughput plant phenotyping.

To ensure that the selected literature was closely aligned with the closed-loop perception–decision–action framing of this review, a set of inclusion and exclusion criteria was established, as summarized in Table 1. The overall screening workflow followed a four-stage PRISMA-style procedure comprising identification, screening, eligibility assessment, and final inclusion, illustrated in Figure 1. After removing duplicates and records clearly irrelevant based on title and abstract, full-text assessment was performed against the eligibility criteria in Table 1. A total of 283 representative studies were finally retained and synthesized to form the technical chapters of this review (Section 2, Section 3 and Section 4).

Because the reviewed literature spans heterogeneous tasks—sensing, SLAM, deep-learning-based phenotypic analysis, manipulation, and edge deployment—a uniform quantitative scoring scheme is not appropriate. Instead, a qualitative quality appraisal was performed along four dimensions: (i) clarity and completeness of the platform, sensor, and algorithmic description; (ii) the realism of the validation environment (controlled indoor, greenhouse, or open field); (iii) the adequacy of evaluation metrics relative to the task (e.g., mAP/IoU for detection and segmentation, RMSE/$R^{2}$ for trait regression, trajectory error and success rate for navigation and manipulation); and (iv) the reproducibility of the reported results, including availability of datasets, code, or hardware specifications. Studies validated under realistic field conditions with well-reported metrics were given greater weight in the synthesis, whereas works with limited validation were retained only when they contributed methodological novelty. This appraisal is not used to rank individual studies, but rather to contextualize the generalizability of the findings discussed throughout the remainder of this review.

## 2. Mobile Phenotyping Platforms

**Figure 2:**
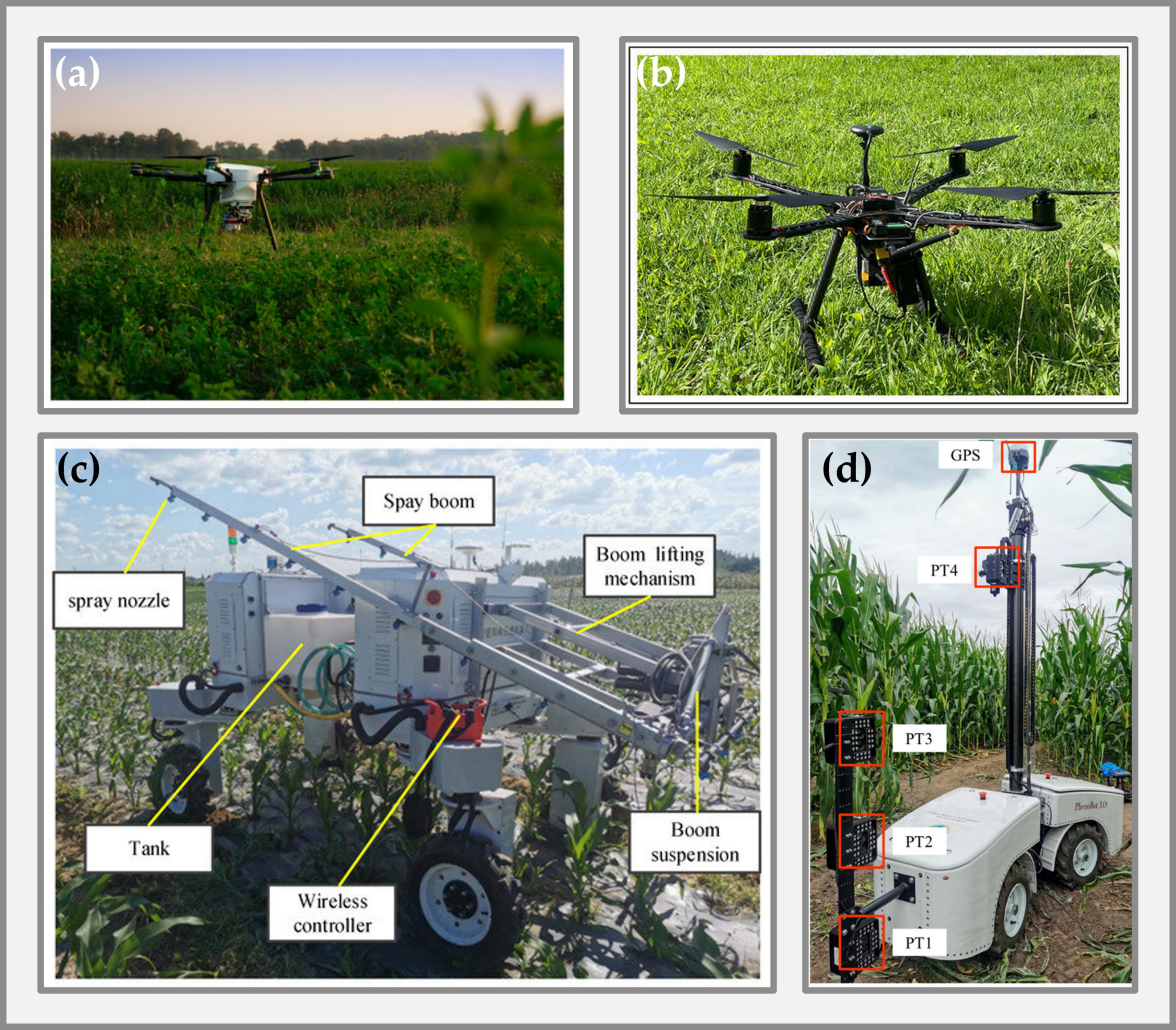
Representative examples of mobile plant phenotyping platforms under field conditions: (**a**) [46] and (**b**) [47] are UAV platforms; (**c**) [48] and (**d**) [49] are UGV platforms. (**a**) Reprinted from Ref. [46] under a CC BY 4.0 license. (**b**) Reprinted from Ref. [47] under a CC BY 4.0 license. (**c**) Reprinted from Ref. [48] under a CC BY-NC-ND license. (**d**) Reprinted from Ref. [49] under a CC BY 4.0 license.

With the rapid development of high-throughput plant phenotyping, traditional static observation facilities are increasingly unable to meet the demand for high-frequency, multidimensional, and multiscale monitoring of crop growth under open-field conditions. Compared with fixed platforms, mobile phenotyping systems can maintain broad spatiotemporal coverage while flexibly integrating diverse sensor types, thereby substantially improving the efficiency, resolution, and environmental adaptability of field phenotypic data acquisition. At present, the principal mobile platforms used for field-based high-throughput phenotyping are Unmanned Aerial Vehicles (Figure 2a,b) and Unmanned Ground Vehicles (Figure 2c,d). The former are particularly effective for rapid, large-area, non-contact observation, whereas the latter are better suited for high-resolution, fine-grained phenotyping in near-canopy and under-canopy environments. Figure 2 illustrates representative field applications of these two platform types. Notably, although UAVs play an important role in field phenotyping, their main strengths lie in large-scale coverage and rapid canopy-level observation. By contrast, for the close-range sensing, autonomous navigation, environment interaction, and intervention-oriented capabilities emphasized in later sections, UGVs serve as the more direct technical carrier. Accordingly, this section briefly reviews UAV-based platforms, then focuses on the configurations, operational modes, and engineering constraints of UGVs, and finally discusses the system-level potential of UAV–UGV collaboration in high-throughput plant phenotyping.

To strengthen the systems perspective of this section, the comparison below is organized around several common dimensions, including sensing geometry, typical phenotypic outputs, autonomy level, coverage efficiency, calibration and data-alignment requirements, operational constraints, and reproducibility. Under this lens, UAVs are primarily considered as large-area canopy-observation platforms, UGVs as close-range robotic carriers for near-canopy and under-canopy phenotyping, and UAV–UGV collaboration as a multiscale coordination framework linking broad-area screening with targeted precision measurement.

### 2.1. Unmanned Aerial Vehicles (UAVs)

As non-contact remote sensing platforms, UAVs have become one of the most representative mobile solutions for field crop phenotyping because of their rapid deployment, broad coverage, and ability to carry multiple sensing payloads. Compared with manual ground surveys, UAVs can acquire data over large plots or even farm-scale areas within a short time, effectively overcoming the spatial and temporal limitations of conventional approaches [45]. They are therefore particularly well suited to periodic monitoring at key growth stages and to the rapid extraction of macro-scale phenotypic traits such as canopy structure, population vigor, and stress response [45,50].

From the standpoint of platform configuration, UAVs commonly used in agricultural phenotyping can be broadly classified into multirotor, fixed-wing, and hybrid types [51,52]. Multirotor UAVs can take off and land vertically and hover in place, allowing flexible operation in spatially constrained field environments and enabling low-speed or stationary image acquisition with high overlap, which is advantageous for improving the geometric accuracy of orthomosaics and 3D reconstruction [53]. However, these platforms are typically constrained by battery capacity, resulting in relatively short flight duration and limited suitability for long-endurance or ultra-large-area missions [44]. By contrast, fixed-wing UAVs rely on aerodynamic lift during cruise and therefore provide greater flight range and coverage, making them more appropriate for continuous farm-scale monitoring and regional crop growth assessment [54]. In recent years, hybrid UAVs that combine the takeoff flexibility of multirotor systems with the cruise efficiency of fixed-wing platforms have attracted increasing attention and are regarded as a promising option for large-scale field phenotyping [55].

The phenotyping capability of UAVs depends to a large extent on the types of sensors they carry. RGB cameras, when combined with photogrammetry and Structure from Motion (SfM) algorithms [56], can reconstruct 3D canopy point clouds from multi-view 2D imagery and support the extraction of phenotypic traits such as plant height, canopy cover, and micro-topography. Previous studies have shown that plant height estimated from UAV-SfM is highly consistent with manual measurements [57]. Multispectral and hyperspectral imaging can capture key spectral bands such as the red-edge and near-infrared (NIR), enabling the calculation of vegetation indices such as NDVI and NDRE to reflect leaf nitrogen status, chlorophyll content, and photosynthesis-related characteristics [58,59,60]. For example, Shao et al. [61] combined UAV multispectral and thermal infrared imagery with machine learning to achieve high-accuracy estimation of aboveground biomass (AGB) in maize. In addition, compared with passive optical imaging, LiDAR provides stronger canopy penetration and can effectively alleviate ground-point loss and the resulting bias in plant height estimation under dense canopy conditions [62].

Despite the substantial gains in throughput offered by UAVs for large-area field phenotyping, they are inherently constrained by both their observation geometry and their task capabilities. First, because UAVs primarily rely on top-view or oblique-view observation, they are highly susceptible to canopy occlusion and therefore limited in their ability to stably capture organ-level or under-canopy traits such as stem structure, lower-leaf posture, ear or fruit morphology, and concealed pest or disease symptoms. Second, their payload capacity, endurance, and onboard computing resources are all constrained by platform size and power systems, which in turn limit their use in continuous close-range observation, complex environmental interaction, and active intervention tasks. Overall, UAVs are better suited to rapid scanning and macro-scale perception over large areas than to high-precision, near-canopy, and long-duration continuous observation when used alone. From a system-evaluation perspective, representative UAV phenotyping studies commonly report metrics such as MAE or RMSE for height-related trait estimation, IoU or F1-score for segmentation tasks, and correlation-based indicators for spectral trait inversion or biomass estimation [56,57,61,63]. In practice, however, UAV performance is strongly influenced by canopy architecture, flight altitude, image overlap, illumination stability, and calibration quality [54,55,57,62]. Moreover, although UAV-mounted LiDAR can improve canopy-surface reconstruction and reduce height-estimation bias under dense canopy conditions, its scan density, payload burden, and cost constraints remain different from those of close-range ground-based LiDAR systems [62,64]. As a result, typical failure modes include canopy-surface bias under dense stands, degradation of 3D reconstruction under insufficient overlap or wind-induced motion, and reduced robustness of trait estimation across variable field conditions [44,57,63].

### 2.2. Unmanned Ground Vehicles

**Figure 3:**
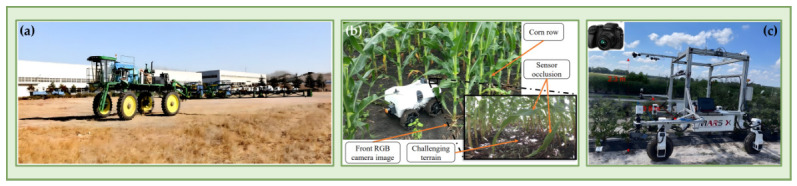
Three typical unmanned ground vehicles for agricultural scenarios. (**a**) High-clearance platform [69]; (**b**) compact inter-row platform [70]; (**c**) modular research platform [71,72]. (**a**) Reprinted from Ref. [69] under a CC BY 4.0 license. (**b**) Reprinted from Ref. [70]. (**c**) Reprinted with permission from Ref. [71]. Copyright 2022, John Wiley and Sons. Reprinted with permission from Ref. [72]. Copyright 2025, Elsevier.

Unlike UAVs, which primarily provide canopy-scale top-view information, ground robots can enter crop rows or move beneath the canopy and acquire high-fidelity phenotypic data from side-view, horizontal-view, or even upward-view perspectives that are closer to target organs [65,66]. As a result, UGVs possess inherent advantages in organ-level trait extraction, 3D structural reconstruction, perception under occlusion, and continuous close-range monitoring, making them the core platform type with greater engineering extensibility in current mobile phenotyping systems [30,67,68]. In terms of mechanical configuration and typical application scenarios, agricultural phenotyping UGVs can generally be categorized into high-clearance cross-row platforms (Figure 3a), compact inter-row platforms (Figure 3b), and modular research platforms (Figure 3c) [30,69,70,71,72]. These different configurations emphasize different trade-offs in traversability, payload capacity, near-canopy sensing mode, and system integration flexibility, together forming a UGV platform spectrum for different crop types, field conditions, and research objectives.

High-clearance cross-row platforms are typically built on tractor-based or specially designed high-mobility chassis and are well suited to phenotyping tall crops such as maize and sorghum. These platforms provide large ground clearance and strong payload capacity, allowing simultaneous integration of multispectral cameras, near-infrared cameras, LiDAR, and other sensors for non-destructive measurement of multiple agronomic traits, including canopy structure, biomass, water status, and lodging characteristics [73]. For example, the BreedVision platform uses a multi-sensor configuration to acquire crop water content, lodging rate, tiller density, biomass, and other traits directly in the field [74]. The main advantages of this platform type are high payload redundancy and strong system stability, which make it suitable for synchronized acquisition of multi-source sensor data, although its large physical size generally reduces maneuverability and flexibility.

Compact inter-row platforms place greater emphasis on traversability in narrow spaces and close-range observation. They can move steadily at low speed within crop rows and approach target organs such as leaves, stems, and fruits from the side or from below the canopy, thereby effectively mitigating the occlusion problems associated with top-view observation [75]. TerraSentia is a representative example of such a compact autonomous ground platform [76]. Equipped with multiple RGB cameras and LiDAR, it can perform high-precision scanning within maize rows and extract latent phenotypic features using deep learning methods [77], supporting plant structure modeling and biomass distribution estimation. This side-view or under-canopy observation mode is especially advantageous for capturing fine-scale phenotypic information, such as leaf number, stem diameter, inter-plant structural variation, and localized disease symptoms, that is difficult to resolve robustly from aerial views [78,79]. Compared with high-clearance platforms, inter-row UGVs are better adapted to organ-level data acquisition and occlusion-rich scenarios and are more closely aligned with the later development of closed-loop perception–decision–action systems.

Modular research platforms are primarily intended for algorithm validation, system integration, and functional extension. Representative examples include Clearpath Robotics’ Husky and Saga Robotics’ Thorvald [64,80]. These platforms typically provide open mechanical and electrical interfaces and are compatible with mainstream robotic software frameworks such as ROS, enabling rapid integration of cameras, LiDAR, robotic arms, and spectral sensors for experimental validation of field navigation, visual recognition, and phenotype extraction methods [81]. Their key strengths lie in high system reliability, strong extensibility, and good experimental reproducibility, which make them widely useful for comparing the effects of different sensor configurations, algorithmic frameworks, and control strategies on phenotyping quality and operational performance [82,83]. Although they may not offer optimal traversability or efficiency for highly specialized agricultural tasks, they are highly valuable for supporting rapid method development and prototyping.

Overall, UGVs substantially expand both the depth and dimensionality of field phenotypic data acquisition through their close-range, multi-view, and high-payload operational characteristics. In particular, for tasks involving organ-level phenotyping, under-canopy structural analysis, long-term continuous monitoring, and the integration of sensing with action, UGVs exhibit stronger engineering suitability than UAVs. From the perspective of advancing high-throughput plant phenotyping toward autonomy, fine-grained observation, and closed-loop operation, UGVs are not only an important component of mobile phenotyping platforms but also the principal carrier of the core technical framework discussed in the following sections. From a systems-taxonomy perspective, these three UGV configurations correspond not only to different chassis forms, but also to different sensing geometries, trait targets, and autonomy requirements [30,64,73]. High-clearance platforms are better suited to row-scale or canopy-level sensing with stronger payload redundancy, whereas compact inter-row platforms are better suited to close-range organ-level observation under occlusion and therefore place stricter demands on row following, localization robustness, and obstacle avoidance [64,75,76]. Modular research platforms, by contrast, are especially valuable for system integration, algorithm validation, and reproducible experimentation because they facilitate flexible sensor mounting and software-hardware co-development [80,81,82,83]. Accordingly, platform selection is best understood as a trade-off among payload capacity, maneuverability, autonomy requirement, and engineering complexity rather than as a simple choice of vehicle size [30,64,82].

### 2.3. Comparative Advantages and Limitations of UAVs and UGVs

From the standpoint of phenotyping requirements, UAVs and UGVs should not be viewed as simple substitutes for one another. Rather, they exhibit clear functional differentiation in terms of spatial scale, observation depth, operational throughput, and environmental interaction. The main strengths of UAVs lie in their high mobility, broad coverage, and relatively simple deployment, which make them suitable for rapid large-area scanning and for extracting macro-scale phenotypic indicators such as canopy height, coverage, population vigor, and thermal stress [53,84]. However, their predominantly top-view or oblique-view observation geometry inevitably introduces occlusion-related limitations. This is particularly problematic in dense, tall crops such as maize and sorghum after canopy closure, where upper leaves often block the middle and lower organs, making it difficult for UAVs to stably capture key agronomic traits such as stem diameter, basal disease symptoms, and ear or fruit morphology [63,85,86,87]. Moreover, although UAVs substantially improve acquisition throughput, their ability to resolve small lesions, early pest infestation, and fine close-range structural details remains limited by flight altitude, endurance, and payload constraints [44,63,88].

In contrast, UGVs can operate much closer to target plants and collect high-resolution data from downward, side, and horizontal viewpoints. Their stronger payload capacity also allows integration of multimodal sensor systems, enabling richer characterization of organ-level and under-canopy traits [22]. In addition, UGVs typically support longer continuous operating times, making them more suitable for continuous or semi-continuous monitoring of localized areas and for the integration of subsequent active intervention tasks. Nevertheless, UGVs also have important limitations. Because they move more slowly and cover less area per day, and because they must address inter-row path planning, autonomous localization, obstacle avoidance, and traversal over complex terrain, their deployment is usually more complicated than that of UAVs. In addition, muddy surfaces, slope variation, and dense canopy obstruction can further affect their motion stability and navigation accuracy [22,44]. Accordingly, the difference between UAVs and UGVs is not merely a distinction between aerial and ground platforms, but rather a distinction between two complementary yet difficult-to-unify task demands in high-throughput plant phenotyping: high-throughput observation for broad-area coverage and rapid screening, and high-information-density observation for fine structural analysis and continuous close-range monitoring. A single platform rarely satisfies both requirements simultaneously. For this reason, UAVs are more appropriate for global perception and regional screening, whereas UGVs are more appropriate as the core carrier for near-canopy precision observation, complex environmental interaction, and closed-loop operation. Table 2 summarizes the key characteristics of the two platform types.

### 2.4. UAV–UGV Collaboration

**Figure 4:**
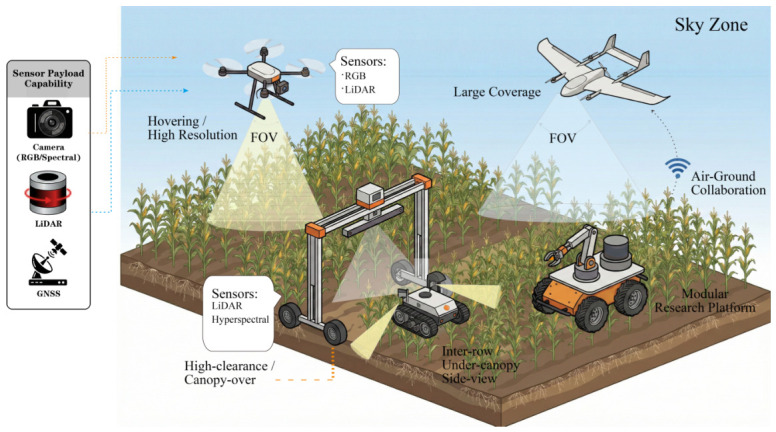
Schematic illustration of the collaborative air–ground framework for high-throughput plant phenotyping. Note: This figure shows the functional division and coordination between aerial and ground mobile platforms in complex field environments. The aerial layer includes multirotor UAVs for high-resolution hovering observation and fixed-wing platforms for rapid large-area coverage, both of which primarily support broad canopy-scale perception. The ground layer includes high-clearance platforms, inter-row platforms, and modular research platforms, which are mainly used for fine-grained acquisition of near-canopy, under-canopy, and organ-level phenotypic information. The dashed arrows indicate the task transition from aerial global scanning to ground-based targeted precision measurement, while the legend on the left summarizes the typical sensor payloads that can be integrated on different platforms, illustrating the multiscale collaborative strategy of “broad-area scanning–local precision measurement” in high-throughput plant phenotyping.

Given the strong complementarity of UAVs and UGVs in terms of coverage efficiency and observation precision, air–ground collaboration has gradually emerged as an important direction in high-throughput plant phenotyping [89]. Its core idea is not simply to combine two platform types, but to construct a task-layered workflow of “global scanning–local precision measurement” through information transfer and functional division (Figure 4). In this framework, UAVs perform rapid large-area scouting, canopy information extraction, and the identification of potentially anomalous regions, while UGVs use aerial prior information to conduct targeted navigation, close-range observation, and even active sampling. In this way, both throughput and data granularity can be balanced under limited time and resource constraints [22,90]. Existing studies have provided initial evidence of the feasibility of this paradigm. For example, Yue et al. [38] proposed the deep learning framework MSRNet based on coordinated UAV–UGV multi-view imaging, integrating UAV top-view canopy imagery with UGV side-view ear imagery, and achieved an overall accuracy of 84.5% in maize developmental stage recognition, representing an improvement of 3.8–13.6% over single-view models. Notably, Tan et al. [91] introduced transfer learning by fine-tuning an object detection model pre-trained on UGV imagery using UAV imagery, and, together with the Segment Anything Model (SAM), proposed an optimized DM-Count model that successfully achieved cotton boll mapping. Similarly, Petti et al. [92] incorporated transfer learning with both UAV and UGV imagery to accomplish phenotyping tasks for cotton flowers, thereby advancing breeding research. These studies indicate that cross-platform multi-view information fusion can improve phenotypic recognition accuracy while also mitigating the information loss associated with single-view observation.

Despite its promise, air–ground collaboration still faces several system-level challenges. Firstly, at the perception level, data collected by different platforms must be aligned spatiotemporally within a unified coordinate system and semantically reconciled to ensure spatial consistency and interpretability in downstream information fusion [93]. Secondly, at the decision level, efficient scheduling and task planning mechanisms are required to ensure that UAVs and UGVs can divide responsibilities appropriately, compensate dynamically for each other, and avoid resource conflicts during collaborative operation [65]. Thirdly, at the execution level, stable inter-platform communication, task allocation, and state synchronization are all critical to overall system efficiency [94]. Looking ahead, developments in multi-UAV formations, heterogeneous robot teams, 5G communication, and distributed control are expected to further improve continuous sensing and dynamic management over large-scale agricultural fields [95,96]. Nevertheless, from the perspective of the technical focus of this review, air–ground collaboration is better understood as an extensible framework for large-scale phenotyping systems, whereas the core platform that truly carries the closed-loop capabilities of close-range sensing, autonomous decision-making, and environment interaction remains the ground robot.

More specifically, the practical effectiveness of air–ground collaboration depends on whether the end-to-end workflow remains stable across cross-platform sensing, communication, and execution [39,44]. A typical pipeline includes aerial scouting, target-region proposal, spatial and temporal data alignment, ground-task assignment, close-range navigation and sensing, and final trait-level fusion [39,41,44]. In this process, key bottlenecks include frame-transformation or synchronization errors between aerial and ground observations, communication delay or task-update instability during coordination, and semantic inconsistency caused by viewpoint, scale, and occlusion differences across platforms [39,41,44]. These issues explain why UAV–UGV collaboration is not merely a matter of combining two sensing systems, but a systems-integration problem requiring robust calibration, synchronization, task allocation, and error-tolerant fusion [44,47].

## 3. Core Technology: Perception, Decision, and Action

As smart agriculture moves beyond proof-of-concept toward large-scale deployment in real-world settings, high-throughput plant phenotyping is undergoing a fundamental shift from static observation platforms to mobile robotic systems. Throughout this review, the terms “mobile phenotyping system”, “field robot”, and “phenotyping platform” are used interchangeably to refer to autonomous ground-based robotic systems designed for plant phenotyping tasks. Conventional phenotyping platforms have largely relied either on automated facilities in controlled environments or on relatively simple remote sensing approaches. While these systems offer advantages in standardization and throughput, they often struggle to simultaneously achieve environmental adaptability, sensing precision, and sustained operation under complex field conditions. By contrast, modern mobile phenotyping systems designed for open-field environments must do more than acquire plant images or point clouds; they must continuously perform environmental understanding, autonomous mobility, target interpretation, and physical interaction in unstructured settings. Accordingly, the central challenge in high-throughput plant phenotyping is no longer the performance improvement of an individual sensor or algorithm, but the construction of an autonomous closed-loop robotic framework integrating perception, decision-making, and action.

At the system level, this closed loop comprises at least four tightly coupled components. First, the perception layer must simultaneously interpret the environment and plant targets: the former provides geometric and semantic information for localization, mapping, and navigation, while the latter supplies high-spatiotemporal-resolution data for analyzing plant morphology, physiology, and pathology. Second, the decision layer integrates information at two levels, motion planning and phenotypic understanding, so that the robot can operate safely and efficiently in complex field environments while converting multisource observations into interpretable and quantifiable phenotypic traits. Third, the action layer reflects the fundamental advantage of robots over conventional remote sensing platforms: beyond simply reaching and seeing a target, the system can actively improve observation conditions and expand measurable trait boundaries through chassis posture adjustment, viewpoint adaptation, robotic-arm-assisted supplementary imaging, and contact-based operations. Finally, the deployment layer determines whether these capabilities can be translated into field practice, subject to engineering constraints such as edge inference, thermal management, communication, and energy supply. Following this logic, the remainder of this section reviews recent advances in mobile phenotyping robots from the perspectives of perception, decision-making, action, and deployment. For consistency, these four modules are discussed with attention to their typical inputs and outputs, main field error sources, representative evaluation criteria, and major deployment constraints.

### 3.1. Perception: From Multimodal Observation to Integrated Localization-Aware Sensing

**Figure 5:**
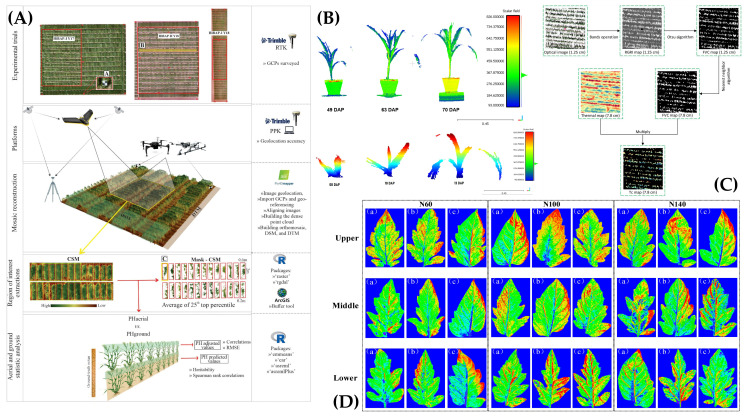
Common sensing modalities used in mobile phenotyping platforms and their corresponding trait analysis tasks. (**A**) RGB/RGB-SfM: Used for canopy morphology characterization and 3D crop surface reconstruction, supporting extraction of canopy coverage, organ counts, and plant height [105]. (**B**) LiDAR: Provides penetrative 3D point clouds for canopy structure, organ geometry, and plant spatial architecture analysis [106]. (**C**) Thermal infrared imaging: Characterizes transpiration, stomatal conductance, and water stress through canopy temperature distribution [107]. (**D**) Hyperspectral imaging: Enables visualization of biochemical indicators such as leaf nitrogen and chlorophyll based on spectral responses at different wavelengths [108]. Reprinted from Refs. [105,106,107,108] under a CC BY 4.0 license.

The perception system is the information entry point of a mobile phenotyping robot, and its role now extends far beyond conventional image acquisition. In real field environments, it must simultaneously fulfill two tasks: environmental perception for the robot itself, which supports localization, mapping, navigation, and obstacle understanding [97,98,99,100], and trait perception for plant targets, which enables analysis of crop morphology, physiological status, and pathological response [101,102,103,104]. This dual requirement means that mobile phenotyping systems cannot rely on a single sensing modality, but instead must improve robustness and information density through the coordinated use of multimodal sensing, 3D reconstruction, and semantic understanding. Figure 5 summarizes representative sensing modalities commonly used in mobile phenotyping, including RGB/RGB-SfM [105], LiDAR [106], thermal infrared imaging [107], and hyperspectral imaging [108], together with their typical phenotyping tasks. A structured comparison of these modalities in terms of key phenotypic traits, spatial resolution, primary limitations, and UGV deployment suitability is provided in Table 3.

From the perspective of trait perception, visible-light sensing remains the most widely used foundational modality because of its high spatial resolution, low cost, and ease of deployment. Two-dimensional RGB imagery serves as a primary data source for tasks such as leaf area index estimation, coverage analysis, crop density assessment, maturity detection, and organ counting [112,117,118]. Through color-space transformations such as HSV, it can also support crop–soil background separation and the identification of macroscopic visual symptoms such as nitrogen-deficiency chlorosis and necrotic lesions [102]. Furthermore, when combined with Structure from Motion (SfM) and Multi-View Stereo (MVS), sequential RGB imagery can reconstruct 3D canopy point clouds, enabling estimation of structural traits such as plant height, biomass, and canopy volume [119]. In addition, some studies have used segmentation algorithms to extract crop shape directly from 2D RGB images [120]. Previous work has shown that low-cost vision-based reconstruction can achieve accuracy comparable to LiDAR for tasks such as plant height estimation. For instance, Madec et al. [121] demonstrated that UAV-SfM can achieve centimeter-level accuracy comparable to LiDAR in estimating plant height and row width, while Fujiwara et al. [122] reported that low-cost vision-based reconstruction was not inferior to RTK-based high-cost solutions for predicting maize height across growth stages. Nevertheless, as a passive imaging modality, RGB sensing is highly sensitive to illumination changes, shadow occlusion, and canopy closure, and is particularly limited in reliably capturing internal structures within dense canopies [109].

Compared with RGB, LiDAR provides a more stable physical basis for 3D structural analysis. By actively emitting laser pulses and measuring their time of flight (ToF), LiDAR acquires high-precision 3D point clouds independently of ambient lighting, making it generally more robust than purely vision-based methods under strong sunlight, shadows, and repetitive textures in agricultural fields. More importantly, LiDAR has a certain canopy penetration capability and can exploit gaps between leaves to detect stems, branches, and the ground surface, thereby alleviating the missing ground-point problem common in photogrammetry [121,123,124,125,126]. For example, Husin et al. [124] and Bates et al. [125] showed that both ground-based and aerial LiDAR can effectively capture internal canopy structure for applications including health monitoring, plant height estimation, and yield prediction. In addition to geometric coordinates, modern LiDAR systems often record return intensity, which can be used to distinguish plant organs based on differences in near-infrared reflectance and thereby support stem–leaf separation and the extraction of fine structural traits such as branch angle and stem diameter [127,128]. Su et al. [129] used a Velodyne VLP-16 LiDAR to collect maize point clouds and achieved high-accuracy phenotypic parameter measurement through projection-based segmentation and fitting algorithms. At the same time, because conventional mechanically rotating LiDARs are vulnerable to reliability issues under high-vibration conditions, solid-state and semi-solid-state LiDARs based on MEMS or Flash technologies are gradually becoming important configurations for agricultural ground robots [110].

If RGB and LiDAR primarily address structural perception, spectral and thermal imaging further extend the ability of mobile phenotyping systems to sense plant physiological status. Multispectral imaging typically includes a small number of discrete bands, such as blue, green, red, red-edge, and near-infrared, among which the red-edge band is particularly sensitive to chlorophyll variation. As a result, it is widely used to compute vegetation indices such as NDVI and NDRE for inferring crop nitrogen status, photosynthetic efficiency, and early senescence [111,112,130]. Hyperspectral imaging, by contrast, provides hundreds of contiguous bands and can capture much finer spectral signatures, supporting the inversion of more complex biochemical constituents such as anthocyanins, carotenoids, and leaf water content, while also showing distinct potential for early disease detection and stress-resistance screening [113,131,132,133,134]. For instance, Brown et al. [135] showed that hyperspectral leaf reflectance can identify infected soybean plants before visible symptoms appear and can reveal key physiological changes related to chlorophyll and water content. However, the very high dimensionality of hyperspectral data substantially increases storage, transmission, and real-time processing costs, and therefore typically requires edge-side dimensionality reduction and feature selection strategies [114,136,137]. Thermal infrared imaging, meanwhile, characterizes transpiration and stomatal conductance through canopy temperature distribution and plays an important role in plant water-stress detection, drought-tolerance screening, and precision irrigation decision-making [112,115,138,139].

For mobile phenotyping robots, perception is not only about observing plants but also about understanding the surrounding environment and determining the robot’s own position. Because real field environments frequently involve GNSS occlusion, abrupt illumination changes, repetitive textures, and terrain-induced disturbance, localization-aware sensing has become a foundational capability for system autonomy [140]. Although RTK-GNSS can provide centimeter-level absolute positioning in open areas, satellite signals often degrade or fail in tall-crop fields and orchards because of canopy blockage and multipath effects [141]. Consequently, modern agricultural robots increasingly rely on fused localization frameworks that integrate wheel odometry, inertial measurement units (IMUs), and visual or LiDAR data, with the IMU providing high-frequency attitude estimation and short-term dynamic compensation, while external sensors correct long-term drift [141,142,143].

**Figure 6:**
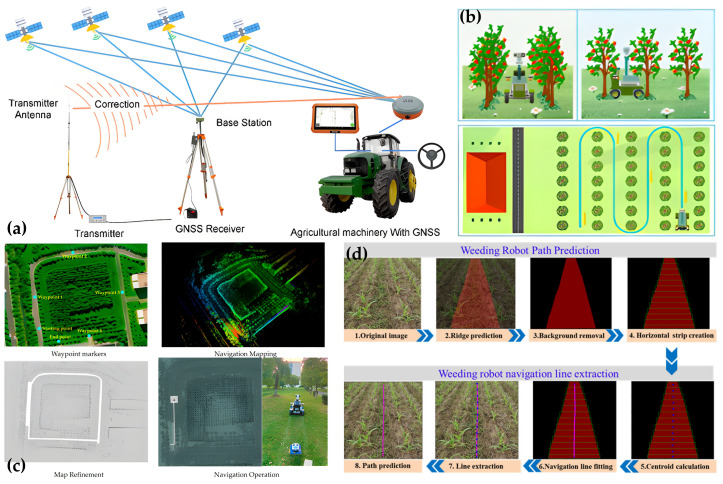
Schematic illustration of operating strategies for mobile phenotyping robots in unstructured field environments. (**a**) Principle of GNSS-RTK positioning [141]. (**b**) GNSS-RTK-based navigation of an agricultural robot in an orchard environment [142]. (**c**) LiDAR-based mapping, path planning, and navigation operation [142]. (**d**) Navigation-line extraction based on semantic segmentation [152]. (**a**) Reprinted from Ref. [141] under a CC BY 4.0 license. (**b**,**c**) Reprinted from Ref. [142] under a CC BY 4.0 license. (**d**) Reprinted from Ref. [152] under a CC BY 4.0 license.

In this context, SLAM has become a key technology for autonomous navigation in agricultural fields. Because lighting conditions in agricultural environments are highly unstable, purely visual SLAM tends to degrade under strong direct sunlight, shadow transitions, and repetitive textures. By contrast, LiDAR SLAM methods such as LOAM, LIO-SAM, and the FAST-LIO family are less dependent on illumination and can exploit geometric priors such as crop-row structure for feature matching, resulting in greater robustness in complex field environments [142,144]. For inter-row navigation, LiDAR point clouds can be used to accurately extract crop-row lines and construct local occupancy maps to support path planning [145,146]. Building on this, an important recent trend is the shift from purely geometric mapping to semantic mapping, in which object detection or semantic segmentation networks are incorporated into SLAM so that point clouds or image regions can be assigned labels such as crop, weed, ground, and obstacle [147,148]. For example, Rapado-Rincon et al. [149] proposed the use of specific reference plants as semantic landmarks to support long-term re-localization and growth tracking across repeated inspections. Qu et al. [142] combined SegFormer-based semantic segmentation with visual SLAM in greenhouse environments to construct a real-time semantic point-cloud map for safe path generation and precise navigation, while Pan et al. [150] developed a perception and semantic mapping method for orchard robots that significantly improved navigation performance in complex environments. More importantly, such semantic maps are no longer used solely for navigation; they are increasingly coupled directly with phenotypic analysis. For instance, Zheng et al. [151] proposed a multi-RGB-D SLAM framework that constructs maps containing both geometric structure and semantic labels, enabling centimeter-level 3D reconstruction and apple phenotypic parameter extraction in large-scale orchards. Figure 6 and Table 4 together summarize representative localization and navigation methods, illustrating both their operational principles and comparative performance under field conditions, and showing the evolution of agricultural robot localization from dependence on absolute positioning toward robust environment-understanding-driven localization.

Although single sensors can already support some phenotyping tasks, the combined effects of field occlusion, background clutter, and environmental variation mean that highly reliable phenotyping systems ultimately require multimodal fusion and active vision. The central goal of multimodal fusion is to exploit complementarity across sensing modalities to compensate for the inherent limitations of any single sensor. Recent studies in multimodal representation learning have demonstrated that integrating heterogeneous data sources can significantly improve robustness and semantic understanding in complex environments [153,154,155,156]. These advances suggest that multimodal perception architectures originally developed in computer vision and affective computing may also provide useful methodological references for agricultural phenotyping systems. For example, counting green fruits from RGB images alone is highly susceptible to interference from background leaves, whereas incorporating depth information through RGB-D fusion can substantially improve foreground–background separability [157,158]. Wang et al. [116] proposed the RD-SSD model, which fused RGB and depth images to successfully identify tomatoes occluded by leaves or partially overlapped with neighboring fruits. Similarly, RGB–thermal fusion exploits differences in thermal inertia between fruits and leaves to provide an additional contrast channel for target detection under shadowed or nighttime conditions [159]. In addition, Neural Radiance Fields (NeRF) represent a highly promising direction [160]. The multimodal NeRF framework proposed by Chopra et al. [161] further enhanced target–background separation under complex lighting through cross-spectral scene modeling. At the methodological level, fusion strategies typically include data-level fusion, such as directly inputting depth as an additional channel, and feature-level fusion, in which features are extracted separately from different modalities and then combined. Existing studies indicate that multimodal fusion networks specifically designed for agricultural scenarios can significantly improve detection accuracy for small and occluded targets. For example, Li et al. [36] proposed YOLO-MSRF, which improved mAP50 by 2.3% and mIoU by 3.6% while maintaining good real-time performance; Huang et al. [37] proposed CornMFN, which fused image and meteorological data through cross-attention and improved maize phenological stage recognition accuracy by 6.91% relative to single-modality models.

In complex canopy scenes, passive perception alone is often insufficient to fully resolve severe occlusion, and active vision is therefore becoming an increasingly important complement in mobile phenotyping systems. On the one hand, amodal segmentation learns the complete contour of a target under occlusion and can infer the hidden shape of partially visible objects, thereby reducing bias in fruit counting and size estimation [162,163,164,165]. For example, Kim et al. [162] developed a Transformer-based amodal segmentation network that significantly reduced error in fruit size estimation; Yang et al. [163] proposed the ACBET network, which effectively recovered complete tomato shape under occlusion; and Jiang et al. [165] introduced an amodal segmentation framework for heavily occluded soybean pods based on an improved Swin Transformer and SimAM attention, achieving high-accuracy estimation of seeds per pod. On the other hand, when missing information cannot be fully recovered from single-frame inference, robots can actively change their viewpoint through viewpoint planning. In the so-called Next-Best-View (NBV) strategy, the robot drives the chassis or manipulator toward observation positions with higher expected information gain based on unknown regions or entropy distributions in the current 3D model, thereby acquiring supplementary images and progressively refining target reconstruction [158,166]. This shift indicates that perception in mobile phenotyping systems is evolving from passive sampling toward active information acquisition through perception–action coupling.

**Figure 7:**
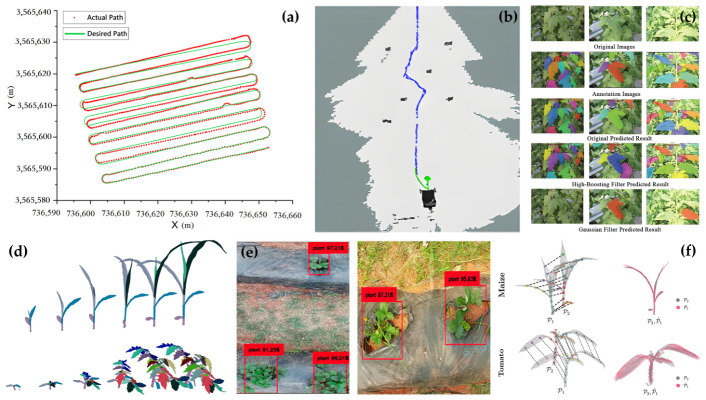
Representative studies illustrating the dual decision-making roles of motion planning and target understanding in mobile phenotyping systems. (**a**) Field path planning and actual trajectory tracking, showing full-coverage decision-making for inter-row operation and headland turning [167]. (**b**) Dynamic obstacle-avoidance trajectories of an agricultural robot in cluttered environments, demonstrating local environmental perception and real-time path replanning [168]. (**c**) Leaf instance segmentation in greenhouse scenes with complex backgrounds, reflecting plant recognition capability based on detection and segmentation [169]. (**d**) Temporal changes and organ labels in 3D point clouds of tomato plants, illustrating the process of temporal 3D phenotypic analysis [170]. (**e**) Real-time strawberry detection on a greenhouse mobile robot, demonstrating end-to-end recognition on edge devices [171]. (**f**) Point-cloud matching and organ correspondence across time points, indicating that 4D registration can support growth tracking and organ-level dynamic phenotypic analysis [172]. Reprinted from Refs. [167,168,169,170,171,172] under a CC BY 4.0 license.

Across these sensing modalities and localization strategies, a clear functional hierarchy has emerged in field practice. RGB-based methods remain the dominant choice for organ-level counting and structural estimation tasks owing to their low cost and deployment convenience [109,157], but their reliance on passive illumination renders them inherently fragile under variable field lighting and in dense-canopy scenarios. LiDAR has established itself as the most robust modality for 3D structural analysis, particularly for traits requiring canopy penetration or illumination-invariant measurement; the progressive transition from mechanically rotating to solid-state designs is reducing the deployment barrier of LiDAR on UGV platforms [110]. Spectral modalities occupy a complementary niche: multispectral imaging is well-suited for continuous canopy-level physiological monitoring, while hyperspectral systems, despite their superior biochemical sensitivity, remain primarily suited to targeted spot-sampling on UGVs owing to high data dimensionality and edge processing costs [114]. For localization, LiDAR SLAM-based approaches have increasingly displaced purely visual methods as the field standard, driven by their robustness to the illumination instability and repetitive textures inherent in crop-row environments [141,142]. The prevailing trend across both sensing and localization is convergence toward multimodal fusion rather than reliance on any single modality [159], reflecting a shared recognition that no single sensor adequately covers the full range of conditions encountered in real agricultural field deployments. Figure 7 further illustrates how these perception capabilities translate into concrete decision-making applications, spanning motion planning, phenotypic analysis, and temporal 3D reconstruction.

### 3.2. Decision: From Motion Planning to Phenotypic Understanding

#### 3.2.1. Motion Planning and Navigation

From the perspective of motion decision-making, robot movement in agricultural fields is not merely a geometric navigation problem, but a joint optimization problem involving geometric constraints, dynamic effects, and agronomic requirements. The primary objective of global planning is typically to cover the entire field while minimizing non-working travel, energy consumption, and soil compaction [173]. Algorithmically, the field is often abstracted as a directed graph, and coverage path planning can be formulated as graph traversal or optimization problems such as the Traveling Salesman Problem (TSP). Graph-theoretic studies on path embedding and Hamiltonian properties provide theoretical insights into how feasible traversal paths can be constructed under structural constraints [174,175]. For example, Bochtis et al. [176] adopted this strategy to address agricultural coverage planning. Because agricultural vehicles are constrained by minimum turning radii, headland turning often requires specially designed trajectories, such as Ω-shaped or fishtail patterns, to balance operational efficiency and maneuverability [177]. Local planning, by contrast, is responsible for handling local disturbances and temporary obstacles during execution of the global path. Yang et al. [178] proposed a multi-objective optimization framework combining an improved A* algorithm with a CNN, in which A* uses NDVI maps to guide machinery away from dense vegetation, while the CNN predicts the trajectories of people or vehicles from real-time LiDAR data, thereby jointly optimizing path length, smoothness, and safety in complex field environments. To address energy consumption and soil protection, Vahdanjoo et al. [179] further proposed 3D coverage-planning models such as Soil2Cover, which directly reduce non-working travel and soil compaction damage through optimization of the path graph structure.

If global planning determines where to go and how to achieve complete coverage, then inter-row straight-line navigation and trajectory correction determine whether the robot can move stably through narrow crop rows. In high-density planting scenarios, even small lateral deviations may cause crop damage or collisions, making crop-row centerline extraction and heading-error correction essential. Current approaches include both traditional machine-vision methods, such as the Hough transform and RANSAC [180,181,182,183], and deep-learning-based semantic segmentation methods [145,184]. To cope with missing plants, broken rows, and partial occlusion, studies often incorporate Kalman filtering or particle filtering to smooth row-line estimation and improve the stability of continuous navigation [145,184,185,186]. At the same time, wet and muddy field surfaces can cause wheel slip, leading to significant discrepancies between commanded and actual motion. Advanced control systems therefore increasingly incorporate slip-rate estimation into the kinematic model by inferring slip in real time from differences among wheel speed, IMU acceleration, and vision- or radar-based velocity, and then actively compensating steering deviations using Model Predictive Control (MPC) or Active Disturbance Rejection Control (ADRC) [100,187,188]. In essence, these methods indicate that motion decision-making in agricultural robotics is evolving from simple geometric centerline tracking toward robust control that explicitly accounts for ground interaction.

Dynamic obstacle avoidance further highlights the distinctive nature of decision-making in agricultural environments. Unlike standard mobile-robot settings dominated by rigid obstacles, field environments often contain both rigid objects, such as rocks, stumps, and farm machinery, and flexible objects, such as extended leaves, weeds, and vines [168,189]. For the former, the robot generally needs to stop or detour, whereas for the latter, direct traversal may in some cases be more efficient. As a result, agricultural robots increasingly require semantic traversability analysis, meaning that they must not only detect the presence of objects ahead, but also infer their physical properties, traversability, and operational risk, and assign different inflation radii and cost weights in the cost map accordingly, so as to generate safe paths better aligned with real operational needs [190,191].

#### 3.2.2. Deep Learning-Based Phenotypic Analysis

Running in parallel with motion decision-making is a second major thread: deep-learning-based phenotypic analysis decision-making, whose core goal is to convert images, point clouds, and temporal observations into quantitative indicators with agronomic significance. Table 5 provides a structured comparison of representative deep learning paradigms across task category, annotation requirements, edge inference feasibility, and critical limitations identified in field deployment contexts. At present, deep learning has become the dominant paradigm in this domain. Single-stage detectors represented by the YOLO family are widely used for real-time counting of fruits, flowers, and similar targets because of their fast inference speed and ease of deployment [192,193,194,195,196,197,198,199,200,201,202,203]. Liu et al. [204] also confirmed, through a systematic comparison across multiple crop fruit-recognition tasks, the suitability of YOLO-based methods for online perception in agricultural robots. For finer-grained tasks, such as leaf-area measurement, lesion-area estimation, or segmentation of complex boundaries, instance segmentation remains a key tool, with Mask R-CNN and its variants serving as representative methods [205,206,207]. For example, Barreto et al. [208] achieved end-to-end extraction of leaf area and disease severity from UAV imagery. To address leaf overlap and cluttered backgrounds, recent studies have increasingly emphasized boundary enhancement mechanisms, significantly improving the segmentation of adhered leaves through edge-aware feature fusion [209] or local refinement modules [169]. For disease grading and cultivar identification, lightweight networks such as MobileNetV3 and EfficientNet, combined with attention mechanisms such as SE and CBAM, have also shown strong potential for early agricultural diagnosis [210].

When phenotyping tasks extend from 2D image analysis to 3D plant structural reconstruction, the decision problem correspondingly shifts from target recognition to structure interpretation. In 3D point-cloud processing, networks such as PointNet++ [211] and PointSegNet [212] can perform point-wise semantic classification, assigning labels such as stem, leaf, or ground to each point. On this basis, clustering can then be used to complete instance segmentation, enabling automated measurement of organ-level traits such as leaf length, leaf width, leaf angle, and stem diameter [213,214,215,216]. To further quantify branching topology, researchers often simplify point clouds into skeletal representations. More recently, Denoising Diffusion Probabilistic Models (DDPMs) have also been introduced into skeleton extraction, allowing systems to recover missing connections and reconstruct more complete topological structures based on learned plant morphological priors, even when point clouds are severely incomplete [217,218]. More importantly, plant phenotypes are inherently dynamic rather than static. As a result, 4D reconstruction and temporal tracking are becoming increasingly important research directions. By registering 3D models acquired at multiple time points, researchers can construct cross-temporal 4D point clouds and use Transformer-based spatiotemporal models to learn plant growth patterns, thereby enabling missing-time-point completion, growth-trend prediction, and even yield estimation [102,219,220].

It is also important to note that conventional deep-learning models typically rely heavily on manually annotated data, whereas agricultural annotation is costly, category-diverse, and subject to strong environmental variation, making model generalization a recurring bottleneck [221]. In this context, foundation models and generative artificial intelligence are beginning to reshape the technical landscape of phenotypic analysis decision-making. Applications of the Segment Anything Model (SAM) in agriculture have shown that large-scale pretrained segmentation models can effectively alleviate annotation bottlenecks in open-set scenarios and improve segmentation generalization in agricultural environments [222,223,224,225]. Ongoing efforts to adapt SAM to agriculture, such as WheatSAM [226] and text-prompted segmentation frameworks combined with Grounding DINO [227], have further demonstrated the potential for crop-organ segmentation under zero-shot or few-shot conditions. At the same time, generative models such as Stable Diffusion and GANs are increasingly being used to mitigate agricultural data scarcity and class imbalance. In 2D applications, Muhammad et al. [228] used diffusion models to generate leaf images with specific disease characteristics to alleviate sample insufficiency in disease recognition. In 3D applications, Roggiolani et al. [229] further explored the generation of synthetic plant point clouds with realistic geometric characteristics for model pretraining and robustness enhancement. Overall, AIGC is gradually evolving from a data-augmentation tool into an important enabling technology for improving the generalization capability of agricultural vision models [162].

**Table 5:** Comparison of deep learning methods for phenotypic analysis in mobile phenotyping systems.

Task Category	Representative Methods	Annotation Requirements	Inference Speed	Deployment Feasibility	Key Limitation (as Reported in the Literature)
Object detection	YOLO family (v4–v12) [193]	Moderate (bounding boxes)	Fast; suited to real-time field deployment	High	Performance subject to target scale, overlap, and canopy background complexity [204]
Instance segmentation	Mask R-CNN and variants [205]	High (pixel-level masks)	Slower than single-stage detectors	Medium	Annotation cost is high; boundary accuracy challenged by leaf overlap and cluttered backgrounds [169,208]
3D point cloud analysis	PointNet++, PointSegNet [211,212]	High (point-wise labels)	Moderate	Medium	High-quality dense point clouds required; agricultural point-wise annotation is costly and expertise-dependent [230]
Foundation models (SAM-based)	SAM, Grounding DINO + SAM [222,227]	Low (zero/few-shot capable)	Moderate	Medium	Model size and computational burden present dual challenge for edge deployment; domain adaptation required for agricultural fine-grained tasks [231]
Generative AI (augmentation)	Stable Diffusion + LoRA/ControlNet, GANs [228,232]	Low (generation-side)	Offline application	Low (offline)	Domain gap between synthetic and real field images limits contribution to generalization; used as complementary rather than primary data source [232]
Temporal/4D spatiotemporal	Transformer spatiotemporal models, DDPMs [217]	Very high (multi-time-point labels)	Computationally intensive	Primarily offline	Requires consistent multi-temporal acquisition; cross-temporal registration and data alignment impose substantial cost [172]

Viewed collectively, the deep learning landscape for phenotypic analysis reveals two intersecting trends. On the detection and segmentation side, YOLO-family detectors have achieved a strong performance–efficiency balance and represent the current standard for real-time field deployment [204], while instance segmentation methods remain bottlenecked by annotation cost and edge computational constraints [233]. The arrival of foundation models such as SAM has substantially reduced the annotation barrier in open-set scenarios [222,231], but their model sizes and inference latencies remain incompatible with direct deployment on power-constrained UGV edge hardware without significant compression [234,235]. Generative AI offers a promising route to alleviating data scarcity, yet the domain gap between synthetic and real field images continues to limit its contribution to model generalization in practice [232,236]. For 3D and temporal tasks, the shift from single-frame point cloud analysis toward 4D spatiotemporal modeling represents the most significant methodological frontier, but simultaneously imposes the heaviest requirements on acquisition consistency, annotation effort, and computational resources—constraints that have yet to be systematically resolved for field-scale deployment [172]. Overall, the field is converging toward a layered deployment strategy in which lightweight detectors handle real-time online inference, while foundation models and 4D spatiotemporal methods are reserved for offline post-processing and deeper phenotypic interpretation.

### 3.3. Action: From Stable Mobility to Active Intervention

If perception and decision-making determine whether a robot can understand its environment and targets, then the action layer determines whether that understanding can be translated into behavior in the physical world. For mobile phenotyping systems, action is not limited to moving the chassis from one location to another; rather, it encompasses a broader set of physical interaction capabilities, including terrain adaptation, posture stabilization, viewpoint adjustment, target contact, and active intervention. It is precisely at this level that the distinctive value of ground robots, relative to traditional UAV-based remote sensing or fixed platforms, becomes fully evident.

The chassis system first determines which operating environments the robot can enter and with what level of stability it can perform observation tasks. Wheeled chassis are structurally simple, energy efficient, and easy to maintain, making them well suited for rapid movement in relatively flat and dry fields. Designs with four-wheel independent steering can further enable zero-radius or small-radius turning, thereby significantly improving maneuverability in narrow headland environments [237,238,239]. Tracked chassis, by contrast, provide larger ground-contact area, lower ground pressure, and stronger traction, and therefore offer better traversability and stability in complex environments such as wet soft soils, paddy fields, or steep orchards [100,240]. Studies have shown that under combined slope and moisture stress, tracked platforms generally exhibit substantially lower slip rates than wheeled systems and are less prone to immobilization or severe skidding [187,237]. For more extreme uneven terrain or obstacle-crossing requirements, wheel–leg hybrid platforms and quadruped-inspired robots are also beginning to enter agricultural applications. These systems can actively regulate body height and posture to maintain sensor stability and chassis traversability, although their energy consumption, control complexity, and endurance remain major constraints on practical deployment [241,242].

Once the chassis enters complex terrain, an appropriate mechanical configuration alone is not sufficient to ensure stable operation; traction management and vibration isolation at the control level are equally critical. In particular, under muddy or low-adhesion conditions, simple PID control often cannot adequately handle slip. More advanced control frameworks therefore commonly incorporate traction control systems (TCS), which estimate slip rate in real time by comparing wheel speed with actual vehicle speed and dynamically adjust motor torque distribution or differential strategies when slip is detected, thereby maintaining platform stability and trajectory-tracking performance [187,243]. In addition, active suspension systems can be used to isolate high-frequency vibrations and ground impacts, protecting precision devices such as cameras, LiDAR, and spectral sensors and reducing image blur and sensor drift that would otherwise degrade phenotypic data quality [237]. This indicates that chassis control in agricultural robotics is not independent of the sensing task, but is directly linked to observation stability and data reliability.

Beyond stable mobility itself, a more distinctive value of mobile robots lies in their ability to actively modify observation conditions. When fruits, ears, or leaves are occluded within the canopy, robotic arms mounted on the platform can use preliminary perception results to actively adjust the camera viewpoint, capturing supplementary images from side, bottom, or other unconventional perspectives in order to construct more complete 3D models and improve counting and size-estimation accuracy [158,166]. Such strategies, which combine perception and adjustment in real time, effectively incorporate action into the phenotypic information acquisition process, transforming the robot from a passive sampling device into an intelligent system that actively optimizes observation conditions. More importantly, robotic manipulators can also perform controlled physical interaction with the environment, thereby overcoming the limitations of conventional non-contact observation. For example, when fruit is completely occluded by leaves, an end effector can plan a collision-free trajectory under visual guidance, gently move the leaves aside to expose the hidden target for imaging, and then restore them to minimize disturbance to the crop [244]. In essence, this type of interactive perception strategy mimics the action logic of human agronomists during field inspection and can improve data completeness without substantially increasing the risk of crop damage. In addition, certain key traits cannot be reliably obtained through vision alone and instead require contact-based measurement. For example, stem mechanical strength must be assessed through the application of physical force [245]. Likewise, some physiological and biochemical traits of leaves can be measured using robotic-arm-mounted fiber-optic probes such as LeafSpec, which are placed directly against the leaf surface to collect high signal-to-noise spectral data while eliminating ambient light interference [246]. Beyond measurement, robotic manipulators can also perform automated tissue sampling, thereby supplying material for downstream molecular analysis and phenotype–genotype association studies [247]. From a long-term perspective, therefore, the action system of mobile phenotyping robots is not merely an auxiliary module for navigation and supplementary imaging, but a key enabler for shifting plant phenotyping from observational workflows toward interactive and operational paradigms.

### 3.4. Field Deployment: Edge Computing and Engineering Challenges in Agricultural Environments

If the sensing, decision-making, and action capabilities described above cannot operate stably over extended periods in real field environments, their academic value is unlikely to translate into practical utility. For mobile phenotyping robots, deployment is not merely a matter of software transfer, but a systems engineering problem involving computation, thermal management, communication, and energy management. For this reason, field deployability is increasingly becoming a key criterion for assessing the practical potential of agricultural robots.

First, edge computing is a prerequisite for real-time operation in mobile phenotyping systems [248,249]. Agricultural robots often operate under unstable or even absent network connectivity, while simultaneously requiring millisecond-level responses to dynamic obstacles, target detection, and path adjustment. Under such conditions, heavy reliance on cloud computing is generally impractical. Data acquisition, preprocessing, inference, and part of the decision process must therefore be executed locally on edge devices. At present, NVIDIA Jetson platforms, such as Jetson Orin NX and AGX Orin, have become important hardware foundations for edge deployment in agricultural robotics by offering strong AI inference capability at relatively low power consumption (15–60 W) [92,250]. For example, Shuai et al. [251] achieved real-time weed detection in soybean fields on a Jetson Orin platform at 59 FPS while maintaining power consumption at 65 W. Similarly, the GTDR-YOLOv12 model proposed by Yang et al. [252] and the lightweight model developed by Qi et al. [253] both demonstrated effective deployment of Jetson platforms for agricultural vision tasks. Compared with CPU-dominated platforms such as Raspberry Pi 5, Jetson Orin typically provides order-of-magnitude advantages for complex vision workloads and is therefore better suited to the high-throughput inference demands of modern mobile phenotyping systems [254].

However, the adoption of foundation models and high-complexity deep networks also introduces a dual challenge for edge deployment in terms of model size and computational burden, making model compression and lightweight design unavoidable technical directions. Quantization is among the most common solutions, using tools such as TensorRT and Quantization-Aware Training (QAT) to compress model weights from FP32 to FP16 or even INT8 [5,233]. Existing studies have shown that INT8 quantization can typically reduce model size by about fourfold and increase inference speed by 3–8×, while keeping accuracy loss within 1%, making it well suited to real-time field applications [233,255]. Wu et al. [256] further demonstrated that structured pruning can more than double inference speed with almost no loss in accuracy, highlighting the practical value of pruning for agricultural edge devices. Beyond compressing existing models, another important strategy is to design lightweight architectures specifically for edge deployment. For instance, Xu et al. [257] proposed RSCDet, which uses structural optimizations such as RepGhostConv and channel compression to achieve real-time inference at 35 FPS on Jetson TX2 NX while maintaining aphid counting accuracy above 84.5%. Overall, advances in edge computing are driving mobile phenotyping systems from offline analysis platforms toward online closed-loop systems [257,258].

Beyond computation itself, engineering constraints imposed by field environments also profoundly affect system reliability. Among them, thermal management is one of the most common yet underestimated issues. Agricultural robots often operate for extended periods under high temperatures, direct sunlight, and dusty conditions, while high-performance chips generate substantial heat under full load. Without adequate thermal control, the system may throttle or even fail. To address this, existing systems typically combine passive and active cooling strategies, such as using metal enclosures as heat-dissipation structures, installing high-static-pressure industrial fans for forced air cooling, and reducing solar heat load through sunshades or highly reflective coatings [259,260]. At the same time, airflow channels must also be designed for dust and water resistance in order to prevent damage to electronic components from mud and particulate intrusion [259,260]. Communication challenges mainly arise from discontinuous network coverage and heterogeneous data types in large-scale field environments. On the one hand, low-power, long-range communication technologies such as LoRaWAN are well suited for telemetry transmission and status reporting, providing stable low-bandwidth communication over kilometer-scale distances. On the other hand, for video streams or high-throughput phenotypic data, public 5G/4G links are generally preferred. In communication blind spots, systems may switch to low-Earth-orbit satellite links such as Starlink or adopt a store-and-forward strategy based on local buffering and delayed upload to ensure that critical data are not lost [261,262]. This suggests that future communication architectures for mobile phenotyping systems will likely evolve toward multi-link redundancy and task-layered communication design. Finally, energy management directly determines the operating range and endurance of the platform. In addition to high-energy-density lithium batteries, intelligent battery management systems (BMS) are also essential, as they can dynamically regulate the power consumption of sensors and computing units according to task priority, for example by shutting down high-power devices such as LiDAR during standby or low-load periods [240]. For long-endurance missions, hybrid powertrains and lightweight platforms integrated with solar-assisted charging components are also attracting attention, with the goal of achieving a more balanced trade-off between payload and endurance [240]. From an application perspective, the competitiveness of future high-throughput plant phenotyping robots will depend not only on model accuracy or single-experiment performance, but also on their ability to operate continuously, stably, and with low maintenance in real agricultural environments. For practical deployment, key reporting items should include end-to-end latency, onboard power and thermal conditions, communication mode, and data logging strategy for offline reprocessing. In addition, reproducible comparison across studies requires consistent reporting of the hardware platform, precision mode, input resolution, and whether performance was measured online in field operation or offline under controlled conditions.

## 4. Discussion

Despite the substantial progress made in recent years in the perception, decision-making, and action capabilities of mobile phenotyping robots, and despite their encouraging performance in many controlled experiments, a series of unresolved systemic bottlenecks still hinder the transition from laboratory validation to real field deployment. These bottlenecks do not stem simply from insufficient accuracy of individual algorithms or limitations of isolated hardware components. Rather, they are manifested in insufficient model generalization under complex environmental variation, the continued scarcity of high-quality annotations and diverse data resources, the lack of unified standards and reproducibility protocols across platforms, and the persistent gap between current systems and the requirements of large-scale, long-term, unattended operation. In other words, the central challenge in mobile phenotyping has shifted from whether a working prototype can be built to whether a technological framework can be established that is cumulative, comparable, and deployable. The three challenges discussed below are considered from four connected perspectives: root causes, representative mitigation strategies, remaining limitations, and the implications for future evaluation and benchmarking.

### 4.1. Challenges

Firstly, environmental heterogeneity and limited cross-scenario generalization remain the primary obstacles to the practical deployment of mobile phenotyping systems. In agricultural environments, illumination, climate, season, crop scale, observation viewpoint, and platform height all vary continuously, such that high performance achieved under a single condition often does not transfer directly to new fields, seasons, or platform configurations. Ullah et al. [263] pointed out that when a perception system is transferred from a ground vehicle (GV) perspective to a UAV perspective, model performance can degrade markedly due to substantial changes in plant scale, observation geometry, and illumination patterns. Hu et al. [236], in their review of domain adaptation for large-scale agricultural imaging data, further emphasized that the mismatch between training and testing distributions caused by environmental, seasonal, and sensor differences is fundamentally the main reason for the limited generalization of current agricultural vision models. For systems that rely heavily on passive optical imaging, abrupt illumination variation is especially likely to destabilize feature representations and thus degrade recognition and segmentation performance. Therefore, if future mobile phenotyping systems are to achieve cross-season, cross-region, and cross-platform deployment, cross-domain consistency modeling, unsupervised domain adaptation, and robust representation learning must be elevated from optional performance enhancements to core design requirements [236,263,264].

**Figure 8:**
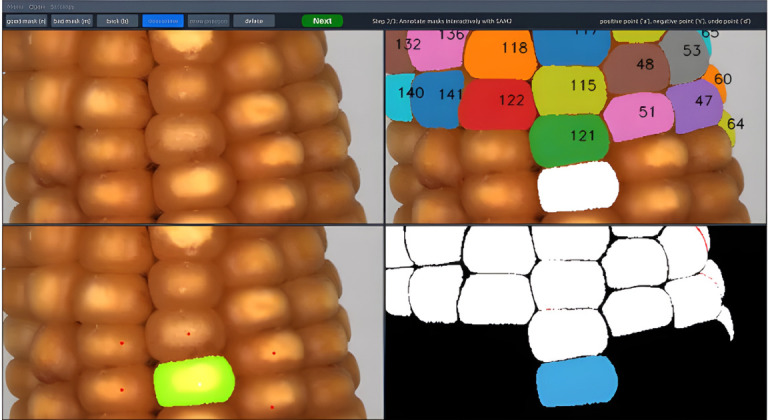
Overview of the ARAMSAM user interface [231]. Reprinted from Ref. [231] under a CC BY 4.0 license.

Secondly, the scarcity of high-quality data and the high cost of expert annotation remain key bottlenecks limiting the continued advancement of agricultural robotic vision models. Unlike general computer vision tasks, high-precision annotation of plant organs typically requires not only pixel-level or instance-level fine-grained delineation, but also annotators with botanical and agronomic expertise, making the data construction process highly dependent on domain experts and significantly increasing both time and economic costs [230]. More broadly, the complexity of agricultural image data and the high cost of annotation are core constraints on the deeper integration of plant science and deep learning [265]. In response, recent studies have begun exploring semi-automatic annotation workflows with expert involvement. For example, Oehme et al. [231] developed ARAMSAM (Figure 8), which uses the automatic mask generation capability of SAM to pre-annotate agricultural images and, in user studies involving experts, significantly reduced the time required for instance segmentation annotation, indicating that foundation-model-assisted annotation can effectively alleviate the scarcity of expert labeling resources. However, improved annotation efficiency alone is insufficient to fundamentally resolve the data problem, because currently available public datasets remain clearly inadequate in terms of scene diversity, seasonal coverage, sensor variety, and environmental complexity [236]. In this context, synthetic data generated by generative artificial intelligence is emerging as an important complementary solution. Hartley et al. [232] proposed generating plant synthetic images for target domains by combining Stable Diffusion, LoRA, and ControlNet. Such methods can preserve key structural information, such as leaf instances, while achieving cross-domain style transfer, and can improve model generalization in complex field environments at relatively low cost by jointly training on real and synthetic data. This suggests that future high-quality agricultural data ecosystems may increasingly depend not on a single source, but on a composite paradigm integrating real data acquisition, foundation-model-based pre-annotation, and generative-model-based expansion.

The lack of standards and the fragmentation of current research are weakening the efficiency of knowledge accumulation in this field. From a systems perspective, the reliability and diagnosability of distributed computing and robotic systems have long been studied through graph-theoretic models of network connectivity and fault tolerance [266,267,268,269,270]. These studies highlight how structural properties of network topologies influence the robustness and diagnosability of large-scale distributed systems. Existing studies on mobile phenotyping differ substantially in platform configuration, sensor parameters, operating conditions, data-processing pipelines, and evaluation metrics, and are often validated under hardware and experimental settings that are specific to individual groups, making results difficult to compare directly. The consequence of this fragmentation is not merely the difficulty of horizontal comparison; more importantly, it obscures the true extent of methodological progress and makes cross-study meta-analysis and cumulative knowledge building extremely difficult. To address this issue, the community urgently needs unified benchmark datasets, evaluation protocols, and metadata standards tailored to mobile phenotyping. At present, MIAPPE (Minimum Information About a Plant Phenotyping Experiment) [271] is the most representative community-driven metadata standard in plant phenotyping and has been adopted by databases such as BreedBase and PHIS as well as the BrAPI interface, providing strong support for the implementation of FAIR data principles. For mobile robotic phenotyping research, the underlying philosophy of MIAPPE is equally valuable, but the current framework still needs to be extended to cover hardware- and system-level metadata, including robot kinematic parameters, platform configuration, sensor mounting arrangements, imaging geometry, and control software versions. Only under such conditions can data from different platforms and laboratories become truly integrable and reusable.

More broadly, standardization should not be limited to unifying data formats; reproducibility should also be treated as a fundamental requirement rather than an added value. One promising direction is the systematic release of standardized annotated datasets, hardware and software configurations, and complete source code for deep learning models [265]. At present, many studies report algorithmic performance metrics but do not provide complete access to model weights, inference code, hardware specifications, data-preprocessing workflows, or deployment details, making it difficult for external researchers to independently reproduce and objectively verify the results. Drawing on established practices in computer vision and genomics, future mobile phenotyping research should at minimum systematically disclose the following: sensor models and calibration parameters, robot chassis kinematic parameters and control software versions, tested model weights and inference interfaces, and complete data-processing pipelines. The reproducibility tiering framework for machine learning in the life sciences proposed by Heil et al. [272] offers a valuable reference in this regard.

### 4.2. Future Perspectives

Beyond these challenges, the future direction of mobile phenotyping can be understood as a staged research agenda. In the near term, progress depends on benchmark construction, dataset pipelines, and reporting standards that improve comparability and reproducibility. In the mid term, the priority is robust generalization under field variation together with uncertainty-aware active observation and adaptive sensing. In the long term, the field is likely to move toward the integration of foundation models, safe embodied policies, and multi-robot orchestration for large-scale autonomous phenotyping.

First, for large-scale breeding trials and long-term field monitoring, single-platform systems and even simple UAV–UGV dual-platform frameworks are approaching their efficiency limits. Multi-robot collectives are therefore likely to become a key pathway for improving throughput, redundancy, and task resilience. For phenotyping tasks spanning hundreds of hectares or more, the system must not only collect data more efficiently, but also tolerate failures, communication interruptions, and local environmental disturbances. Realizing this potential requires treating multi-robot coordination as a genuine systems-engineering problem—one involving decentralized communication, heterogeneous task allocation, conflict resolution, fault tolerance, and fleet-level energy management—rather than simply scaling up single-robot operation. In large-scale distributed computing systems, related issues have long been studied through diagnosability and connectivity analysis in interconnection networks [273,274,275]. Although these theories are not directly transferable to agricultural robotics, they provide useful analytical perspectives once explicitly mapped to communication reliability, failure localization, and coordination robustness in heterogeneous field robot collectives. In parallel, recent advances in real-time industrial communication, such as container-based EtherCAT architectures for virtual PLCs, further suggest that deterministic communication, bounded synchronization error, and cross-platform portability may become important engineering foundations for scalable multi-robot phenotyping systems operating under heterogeneous edge hardware constraints [276]. The MARbLE platform proposed by Boubin et al. [277] showed that, in digital agriculture, multi-robot systems can accelerate task execution and reduce energy consumption through online learning and information sharing when combined with edge-computing nodes and multi-agent reinforcement learning (MARL). Zhu et al. [278] further introduced large language models (LLMs) into UAV-assisted edge-computing networks to enhance multi-agent coordination, enabling adaptive task offloading and cross-region load balancing. Similarly, the COHERENT system proposed by Liu et al. [279] uses a propose–execute–feedback–adjust (PEFA) mechanism to decompose and allocate complex long-horizon tasks in real time to heterogeneous agents, including UAVs, quadruped robots, and manipulators, and significantly outperforms conventional methods in both task success rate and execution efficiency. Reinforcement learning may also become an important future direction for improving adaptive coordination and decision-making in large-scale multi-robot phenotyping systems [280,281]. Together, these studies suggest that future high-throughput plant phenotyping systems are likely to evolve from single-robot platforms into heterogeneous robotic collectives, supported by 5G communication, distributed edge computing, MARL, and LLM-driven high-level task planning [277,278,279,282,283,284,285,286,287,288,289].

Second, the capability boundary of mobile phenotyping robots is expanding from automated data acquisition toward active understanding and adaptive observation. Most current systems still function essentially as mobile sensor carriers: they collect data along predefined routes and then rely on offline algorithms for analysis, while lacking the ability to actively interpret the biological significance of crop status or to dynamically adjust sampling strategies based on immediate observations. Human agronomists, by contrast, do not work in this way. They routinely determine what to inspect next and how to measure it based on local symptoms, historical growth status, and current environmental conditions. Embodied intelligence seeks to create a tighter coupling among perception, cognition, and action, such that robots are no longer merely platforms that transport sensors, but intelligent agents capable of understanding task intent, recognizing biological anomalies, and proactively adjusting their behavior [290,291]. The core logic of this direction can be summarized as a continuous chain of embodied perception, embodied cognition, embodied execution, and embodied evolution, in which multimodal foundation models handle high-level semantic understanding and task planning, while lower-level control networks translate those intentions into executable actions in real time [292]. Within this framework, Vision–Language–Action (VLA) models are widely regarded as one of the most promising technical directions for integrating perception, reasoning, and manipulation in robotic systems. Recent studies have explored adaptive decision-making mechanisms for long-horizon robotic tasks through symmetry-aware or language-guided policies [293]. Such frameworks indicate that multimodal reasoning models may eventually enable agricultural robots to autonomously determine sensing strategies, adjust viewpoints, and perform targeted phenotypic observations. VLA models can directly map natural-language instructions and visual observations into robotic action sequences, thereby giving robots stronger generalization ability for complex long-horizon tasks [294]. However, this capability has so far been demonstrated primarily in general-purpose robotic manipulation settings rather than in agricultural field deployments. In agricultural phenotyping, this suggests that future robots may, in principle, be able to autonomously decide whether to switch sensors, adjust viewpoints, perform additional local scanning, or trigger further operations based on observed leaf chlorosis, abnormal plant architecture, or disease symptoms. At the same time, this prospect is constrained by reality: VLA systems typically require substantial computational and memory resources, whereas mobile agricultural robots face strict constraints in size, power, and thermal dissipation [234,235]. Accordingly, the key challenge for future research is not simply to introduce VLA into agriculture, but to explore how to retain its multimodal understanding and task generalization capability while developing architectures that are lighter, more efficient, and capable of stable edge deployment. It is therefore foreseeable that, provided the aforementioned edge computing and safety constraints are adequately addressed, the deeper integration of embodied intelligence frameworks, multimodal foundation models, and agricultural robotic control systems will enable future mobile phenotyping platforms to gradually evolve from perception systems into integrated cognition–decision–action systems and assume a more proactive role in precision agriculture and crop breeding.

## 5. Conclusions

Ground robots are reshaping the technological boundaries of high-throughput plant phenotyping. Compared with aerial platforms designed primarily for large-area scanning and canopy-scale observation, ground robots can enter crop rows and under-canopy environments to acquire higher-resolution, higher-information-density data through close-range, multi-view, and high-payload operation. This capability extends plant phenotyping from remote observation alone toward autonomous sensing, motion decision-making, and active interaction in complex field environments. This review synthesized the development of mobile phenotyping platforms and core enabling technologies, with particular emphasis on the role of ground robots as the central carrier of closed-loop high-throughput plant phenotyping. The review further provided a structured synthesis of major advances across platform configuration, sensing modalities, localization and mapping, multimodal fusion, motion planning, phenotypic analysis, active observation, robotic intervention, and edge deployment, while identifying the main bottlenecks that continue to limit reproducible and scalable field deployment. The importance of ground robots lies not only in improving acquisition throughput, but also in integrating spatial scale, temporal continuity, environmental adaptability, and physical interaction within a unified technical framework, thereby enabling finer analysis of organ-level traits, under-canopy structures, and dynamic growth processes. At the same time, real-world deployment still depends on overcoming several persistent bottlenecks, including robust generalization under complex field conditions, sustained access to high-quality data and annotations, cross-platform standardization and reproducibility, and the engineering reliability required for long-term unattended operation. As air–ground collaboration, multi-robot systems, embodied intelligence, and foundation models continue to advance, mobile phenotyping platforms are expected to evolve from data-acquisition tools into autonomous systems capable of understanding crop status, organizing observation workflows, and supporting long-term field management. In this sense, high-throughput plant phenotyping will become not only a means of collecting data faster, but also a key link between crop biological understanding, robotic autonomy, and precision agriculture. Among the most pressing unresolved gaps are the absence of cross-domain generalization benchmarks spanning diverse crops, seasons, and sensor configurations; the lack of standardized annotation protocols and open dataset repositories for field-based robotic phenotyping; and the computational incompatibility of current large foundation model architectures with the power and thermal constraints of agricultural edge hardware. Closing these gaps will be essential for translating the technologies reviewed here into reproducible, scalable, and practically deployable field systems.

## Figures and Tables

**Table 1:** Inclusion and exclusion criteria for literature selection.

Criteria	Description
Inclusion	(1) The study focuses on autonomous or semi-autonomous ground-based platforms used for field-relevant plant phenotyping. (2) The work addresses at least one component of the perception–decision–action closed loop (e.g., multimodal sensing, localization/SLAM, motion planning, deep-learning-based phenotypic analysis, active observation, robotic intervention, or edge deployment). (3) Experimental validation is provided using real field, greenhouse, or representative outdoor data, rather than purely simulated results. (4) Peer-reviewed journal or conference papers written in English are prioritized to ensure academic rigor and verifiability. (5) Seminal earlier works are retained when they provide essential methodological or conceptual foundations.
Exclusion	(1) Studies exclusively focused on Unmanned Aerial Vehicles without any air–ground collaboration or ground-robot component. (2) Works limited to static, fully controlled indoor facilities (e.g., conveyor-based phenotyping) without autonomous mobility. (3) Papers reporting only conceptual architectures, patents, or opinion pieces without algorithmic description or experimental evaluation. (4) Duplicated records, non-archival preprints superseded by published versions, and papers lacking sufficient methodological detail for comparative analysis.

**Table 2:** Comparison of UAVs and UGVs in high-throughput plant phenotyping.

Characteristic	UAV	UGV
Operating mode	Aerial imaging from top or oblique view	Ground locomotion with horizontal, side, or upward view
Coverage scale	Large (plot to farm) [84]	Small to medium (plots or inter-row areas) [44]
Primary targets	Canopy-scale traits: structure, height, coverage, thermal stress	Organ-level traits: stems, leaves, ears, fruits
Sensor payload	Limited; lightweight cameras or spectral sensors	High; supports LiDAR, multi-camera, spectrometers, robotic arms
Spatial detail	High resolution but limited to canopy surface	High-fidelity organ-level and structural information
Endurance	Short per mission; battery-constrained	Several hours of continuous operation
Operational efficiency	Fast; suited for rapid large-area screening	Slow; suited for precision close-range measurement
Deployment complexity	Low; mainly flight planning	High; requires navigation, obstacle avoidance, terrain adaptation
Key advantages	High throughput, broad coverage, fast canopy-scale acquisition	High precision, multi-view sensing, fine-grained observation
Key limitations	Poor under-canopy observation; limited endurance and payload [44,63]	Limited coverage; high navigation and terrain demands [22,44]
Autonomy level	Preplanned mission execution [56,57,62]	Teleoperated to fully autonomous depending on system capability [64,76,83]
Main bottlenecks	Georeferencing dependence, occlusion, illumination sensitivity [57,61,63]	Localization drift, row-following, cluttered-field navigation [64,76,81]
Typical outputs	Canopy height, coverage, vigor, thermal traits, biomass proxies [57,61,62]	Stem/leaf/ear morphology, under-canopy structure, 3D close-range traits [73,76,79]
Calibration needs	Radiometric/geometric calibration, flight overlap, temporal consistency [56,57]	Extrinsic sensor calibration, localization consistency, module synchronization [64,76,82]

**Table 3:** Comparison of sensing modalities commonly used in mobile phenotyping robots.

Modality	Key Phenotypic Traits	Spatial Resolution	Main Limitations	UGV Deployment Suitability
RGB/RGB-SfM	Plant height, canopy coverage, organ counting, crop density, maturity [109]	High	Sensitive to illumination changes, shadow occlusion, and canopy closure; limited for capturing internal structures in dense canopy [109]	High—low cost, easy integration, widely validated
LiDAR	3D canopy structure, stem diameter, branch angle, lodging detection [110]	High	Mechanically rotating variants vulnerable to reliability issues under high-vibration conditions; limited to geometric rather than biochemical characterization [110]	High—illumination-independent; solid-state variants increasingly available for agricultural robots
Multispectral	NDVI, NDRE, nitrogen status, chlorophyll content, early senescence [111]	Medium	Limited number of discrete spectral bands; primarily reflects surface-level canopy information [112]	Medium—suitable for canopy physiological monitoring; lightweight and deployable
Hyperspectral	Biochemical constituents (anthocyanins, carotenoids, leaf water content), early disease detection [113]	High spectral resolution	Very high data dimensionality substantially increases storage, transmission, and real-time processing costs; typically requires edge-side dimensionality reduction [114]	Medium—mainly suited for targeted spot-sampling rather than continuous scanning
Thermal Infrared	Water stress, stomatal conductance, transpiration, drought tolerance screening [115]	Medium	Characterizes canopy-level temperature distribution; applicability depends on stable thermal contrast between target and background [115]	Medium—effective for stress screening under controlled observation conditions
RGB-D/Depth fusion	Fruit detection under occlusion, foreground–background separation, 3D shape estimation [116]	Medium–High	Depth channel improves occlusion handling but requires cross-modal registration and calibration; performance subject to sensor alignment quality [36]	High—effective for near-canopy occlusion scenarios; lower cost relative to LiDAR

**Table 4:** Comparison of localization and navigation methods for agricultural ground robots.

Method	Positioning Basis	Illumination Robustness	Terrain Adaptability	Computational Cost	Field Applicability and Constraints
RTK-GNSS	Absolute satellite positioning	Not applicable	High	Low	Centimeter-level accuracy in open areas; signal degrades due to canopy blockage and multipath effects in tall-crop rows and orchards [141]
Visual SLAM	Camera-based feature matching	Low	Medium	Medium–High	Tends to degrade under strong direct sunlight, shadow transitions, and repetitive textures in agricultural environments [144]
LiDAR SLAM (LOAM, LIO-SAM, FAST-LIO)	Laser point cloud geometry	High	High	High	Less dependent on illumination; exploits crop-row geometric priors for feature matching; greater robustness in complex field environments [142]
Semantic SLAM	Semantic object landmarks	Medium–High	Medium–High	High	Enables long-term re-localization using plant-level semantic landmarks; increasingly coupled with phenotypic analysis [149]
Fused (GNSS + IMU + LiDAR/Vision)	Multi-sensor integration	High	High	High	IMU provides high-frequency attitude estimation and short-term compensation; external sensors correct long-term drift; increasingly adopted as standard for agricultural robots [141]

## Data Availability

No new data were created or analyzed in this study.
